# Cardiovascular disease events within 5 years after a diagnosis of breast cancer

**DOI:** 10.1186/s12885-020-06838-w

**Published:** 2020-04-21

**Authors:** Benoîte Mery, Antoine Fouilloux, Elise Rowinski, Judith Catella-Chatron, Jean-Baptiste Guichard, Antoine Da Costa, Fabien Tinquaut, N. Magné, Laurent Bertoletti

**Affiliations:** 1 Department of Medical Oncology, Lucien Neuwirth Cancer Institute, 42270 SAINT PRIEST EN JAREZ, France; 2 Department of Vascular and Therapeutic Medicine, University Hospital of Saint-Etienne, Saint-Etienne, France; 3 Division of Cardiology, Jean Monnet University, Saint-Etienne, France; 4 Public Health Department, Lucien Neuwirth Cancer Institute, St Priest en Jarez, France; 5 Radiotherapy Department, Lucien Neuwirth Cancer Institute, St Priest en Jarez, France

**Keywords:** Breast cancer, Cardiovascular disease, Atrial fibrillation, Cardiovascular prevention

## Abstract

**Background:**

Concern for cardiovascular disease (particularly atrial fibrillation-AF) among women with breast cancer is becoming a major issue. We aimed at determining the incidence of cardiovascular disease events (AF, arterial and cardiac events, venous-thromboembolism-VTE) in patients diagnosed with breast cancer, and assessing potential risk factors.

**Methods:**

We reviewed medical records of all patients diagnosed with breast cancer from 2010 to 2011 in our cancer center. Baseline characteristics of patients and tumors were collected. The main outcome was the occurrence of cardiovascular disease events (AF, VTE, arterial and cardiac events) during the 5-years follow-up.

**Results:**

Among the 682 breast cancer patients, 22 (3.2%) patients had a history of atrial fibrillation. Thirty-four patients (5%) presented at least one cardiovascular disease event, leading to a cumulative incidence of 5.8% events at 5-years ([3.8–7.7] CI 95%), with most of them occurring in the first 2 years. AF cumulative incidence was 1.1% ([0.1–2.1] CI 95%). Factors associated with the occurrence of cardiovascular disease events (including AF) were an overexpression of HER-2 (HR 2.6 [1.21–5.56] *p* < 0.011), UICC-stage III tumors or more (HR 5.47 [2.78–10.76] *p* < 0.001) and pre-existing cardiovascular risk factors (HR 2.91 [1.36–6.23] *p* < 0.004).

**Conclusion:**

The incidence of cardiovascular disease events was 5.8% ([3.8–7.7] CI 95%), with HER-2 over-expression, UICC-stage III tumors or more and pre-existing cardiovascular diseases being associated with them. These findings call for the development of preventive strategies in patients diagnosed with breast cancer.

## Background

Breast cancer is the most common cancer among women worldwide [1]. Longer life spans and decreased rates of breast cancer-specific mortality have been achieved with notably a 5 year-survival of 89% for all stages combined [2]. Such survival rates after a breast cancer diagnosis are higher than they have ever been, due to improvements in cancer therapy. Subsequently, current emerging issues henceforth concern long-term events in survivors. Among these, the magnitude of cardiovascular disease events such as myocardial infarction, stroke or venous thromboembolism (including pulmonary embolism), remains a crucial topic, as they are the leading cause of death worldwide [3, 4]. In particular, cardiovascular disease is now the primary cause of death among patients with breast cancer and remains a major concern [5, 6]. The risk of death from cardiovascular disease following breast cancer is increased in women exposed to cardiotoxic treatments including left-sided radiotherapy, anthracycline-based chemotherapy and trastuzumab [7]. Besides, cardiovascular disease risk factors, including obesity and diabetes may be more present among breast cancer survivors as breast cancer and cardiovascular disease share common risk factors [4, 8].

Another field of cardio-oncology which is increasingly explored, appears to be the increase of atrial fibrillation (AF) incidence in cancer patients [9–11]. Indeed, pre-existing AF and new AF among cancer patients have been significantly highlighted but there is scarce evidence available, especially in breast cancer [12, 13]. Subsequently, there is a crucial need for more epidemiological studies focusing on the link between AF and breast cancer in order to establish both treatment and preventive strategies as the occurrence of AF may affect patients’ prognosis [14]. In parallel, it is well known that breast cancer patients have an increased risk of venous thromboembolism, hence additional concerns as regards the best antiacoagulation strategy for this category of patients with long survival [15]. Furthermore, considering arterial disease, cancer is also a risk factor for ischemic stroke, without full knowledge of best anticoagulant choice. Indeed, most of preventable strategies have been validated in patients without cancer [16]. The objective of the present study was to assess the incidence of cardiovascular disease events (atrial fibrillation, arterial and cardiac events, venous thrombo-embolism) throughout a 5-years follow-up in an unselected consecutive breast cancer cohort and to identify potential risk factors for the occurrence of such events.

## Methods

### Setting and participants

All patients diagnosed with breast cancer between January 2010 and December 2011 at the Lucien Neuwirth cancer institute, depending on the University of Saint-Etienne in France, were included in this retrospective study. Patients with breast cancer recurrence in 2010 and 2011 with a previous breast cancer diagnosis were not included. The Lucien Neuwirth cancer institute is the unique cancer center of the Loire metropolitan area (corresponding to almost 800,000 inhabitants), and concentrates all the patients diagnosed with breast cancer.

A database of all recruited subjects was performed from medical records to collect information related to demographic characteristics, tumor type, tumor grade, histo-pathological characteristics, treatments including surgery, chemotherapy, hormonotherapy or radiotherapy and survival data during 5-years follow up. Data concerning pre-existing cardiovascular disease risk factors including hypertension, hypercholesterolaemia, diabetes or a smoking status were also retrieved, as well as the intake of drugs with cardio-protector effects at the time of initial diagnosis. Calls to each patient’s general practitioner were performed in order to collect such data. A history of AF at time of diagnosis was also examined. Cardiac adverse events associated to therapeutics were scored according to NCI/CTCAE V3.30. The main outcome was the occurrence of AF during therapeutic management and 5-years follow-up. Secondary outcomes were the onset of cardiovascular disease events including arterial or venous thromboembolic diseases and heart failure. The only exclusion criterion was a previous history of malignant disease. This study was approved by the Ethical Committees of the Saint-Etienne University Hospital.

### Statistical analysis

Descriptive statistics were performed, with continuous variables summarized as the mean and standard deviation, and categorical variables presented as numbers and proportions. Comparisons of baseline characteristics were performed using the Student t-test for continuous variables and Fisher tests for categorical variables. Confidence intervals were provided for all incidence figures. Overall survival and progression-free survival were analyzed in accordance with the Kaplan Meier method. Cox proportional hazard regression models were performed. Log-Rank tests were used to analyze risk factors of a cardiovascular event occurrence through univariate analysis. All statistical analyses were completed using the R software. Statistical significance was set at the *P* value < 0.05.

## Results

### Patient characteristics

A total of 682 patients with breast cancer, mostly women (99.2%) were eligible for analysis. Patients were at a mean age of 62.1 years old at cancer diagnosis. The distribution of age was 62.5 (50.9–72.6). The prevalence of obesity was 15.7% (107 patients). A total of 15 patients (2.2%) had metastatic disease at diagnosis while 75 patients (11%) had UICC-stage III tumors. Infiltrating ductal carcinoma was the commonest histological type (80.2%). An overexpression of HER-2 was observed for 78 patients (11.4%). Most tumors (82.8%) were hormone receptor positive. Twenty-eight percent of the patients had a highly proliferative tumor (SBR = 3). A total of 256 patients (37.5%) received adjuvant chemotherapy before radiotherapy. Only the trastuzumab adjuvant was continued during radiotherapy for 60 patients (8.8%). Trastuzumab was preceded of chemotherapy with anthracyclines for 41 patients (68.3%). Almost 50% of patients had pre-existing cardiovascular disease risk factors including diabetes (17.4%), hypertension (73.8%), and hypercholesterolemia (35%). Twenty-two (3.2%) patients had a history of atrial fibrillation. Thirty-one percent of patients were receiving cardiovascular drugs. Patients and tumors characteristics are listed in Table 1. Type of chemotherapy is detailed in Table 2.

**Table 1:** Characteristics of patients (*n* = 682)

	All patients
Patients (n)	682
***Clinical characteristics***	
Gender (female)	676 (99.12%)
Age (mean years)	62.5 (50.9–72.6)
Body weight (mean kg ± SD)	66.3 ± 13.6
Body Mass Index (*n* = 662) (mean kg/m^2^ ± SD)	25.5 ± 5.0
Obesity	107 (15.7%)
***Cancer characteristics***	
Breast side (left)	357 (52.3%)
UICC Breast Staging	
O	39 (0.7%)
I	327 (47.9%)
II	226 (33.1%)
III	75 (11.0%)
IV	15 (2.2%)
Histological type	
Infiltrating Ductal Carcinoma	547 (80.2%)
Infiltrating Lobular Carcinoma	74 (10.9%)
Carcinoma in situ	39 (5.7%)
Histopronostic criteria	
Her2 positive	78 (11.4%)
Hormonal receptors positives	565 (82.8%)
Triple negative	58 (8.5%)
SBR 1–2 grade	454 (66.6%)
SBR 3 grade	191 (28.0%)
***Cancer treatments***	
Surgery	665 (97.5%)
Chemotherapy	256 (37.5%)
Trastuzumab therapy	60 (8.8%)
Radiotherapy	673 (98.7%)
Hormone blocking therapy	551 (80.8%)
***Cardiovascular characterics***	
Cardiovascular risk factors	340 (49.9%)
Hypertension	251 (73.8%)
Diabetes mellitus	59 (17.4%)
Smoking (current or stopped less than 2 years)	53 (15.6%)
Hypercholesterolemia	119 (35%)
Atrial fibrillation (AF) at cancer diagnosis	22 (3.2%)
Cardiovascular treatments at cancer diagnosis	214 (31.4%)
Β-blockers	71 (10.4%)
ACE inhibitor or AIIRAs	100 (14.7%)
CCB	63 (9.2%)
Diuretics	69 (10.1%)
Anticoagulant	20 (2.9%)
One antiplatelet	40 (5.9%)
Dual antiplatelets	3 (0.43%)

**Table 2:** adjuvant cehmotherapy

***N*** = 682		N (%)
Chemotherapy (*N* = 682)	Y	256 (37.5%)
	N	426 (62.5%)
Neo-adjuvant chemotherapy (***N*** = 682)	Y	22 (3.2%)
	N	660 (96.8%)
Adjuvant chemotherapy (***N*** = 682)	Y	234 (34.3%)
	N	448 (65.7%)
Chemotherapy with anthracyclines (***N*** = 256)	Y	174 (68%)
	N	82 (32%)
3 FEC 100–3 DOCETAXEL (*N* = 256)	Y	74 (28.9%)
	N	182 (71.1%)
6 TAC (*N* = 256)	Y	79 (30.9%)
	N	177 (69.1%)
6 FEC (*N* = 256)	Y	1 (0.4%)
	N	117 (45.7%)
4 AC-4 DOCETAXEL (*N* = 256)	Y	19 (7.4%)
	N	237 (92.6%)
4 AC-12 PACLITAXEL WEEKLY: (*N* = 256)	Y	1 (0.4%)
	N	255 (99.6%)
Chemotherapy without anthracyclines (*N* = 256)	Y	82 (32%)
	N	174 (68%)
6 DOCETAXEL-CARBOPLATINE (*N* = 256)	Y	4 (1.6%)
	N	251 (98%)
4 DOCETAXEL-ENDOXAN (*N* = 256)	Y	78 (30.5%)
	N	178 (69.5%)

### Cardiovascular disease events

**Fig. 1:**
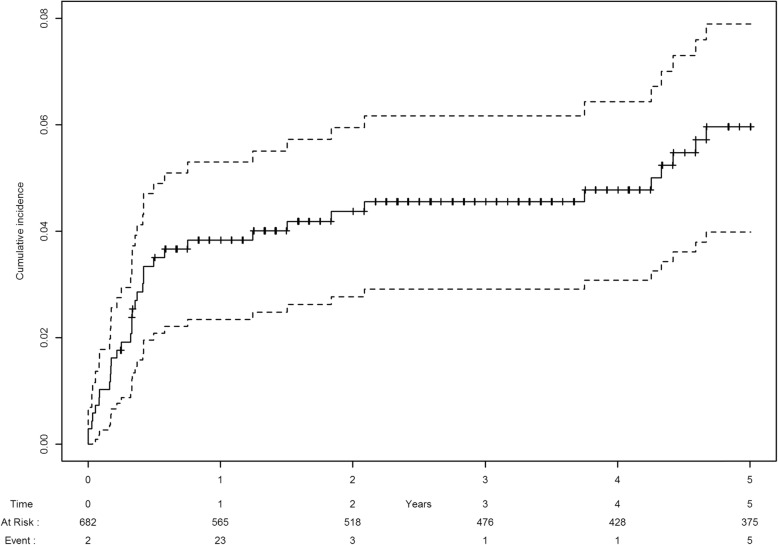
Occurrence of cardiovascular events during follow-up with 95% CI

During the 5-years follow up, 34 patients (5%) presented at least one cardiovascular disease event, with a cumulative incidence of cardiovascular disease events of 5.8% ([3.8–7.7] CI 95%). Five cases of AF were identified, leading to a cumulative incidence of AF of 1.1% ([0.1–2.1] CI 95%), with four receiving DOAC as anticoagulant medication. Venous thromboembolic complications were the most frequent cardiovascular disease event with 4 isolated pulmonary embolism (PE), 12 isolated deep venous thrombosis (DVT), 5 combined PE and DVT and 2 superficial venous thrombosis (SVT). Heart failure has been observed for 3 patients while one had acute myocardial injury. One patient experienced an auriculo-ventricular block III needing pacemaker. Three patients developed ischemic stroke. Two patients were diagnosed with a carotid stenosis. Fourteen patients (2.1%) experimented cardiotoxicity of either anthracyclin, trastuzumab or radiotherapy under treatment, respectively 0.44, 1.2 and 0.44%. Most of the events were heart failure, myocarditis and supraventricular tachycardia other than AF. The mean time from breast cancer diagnosis to the occurrence of AF was 3.6 years whereas it was 1.1 years for cardiovascular disease events other than AF and 1.8 years for venous thromboembolism diseases. Most of the cardiovascular disease events occurred within the first year after diagnosis, with a secondary increase between the 4th and 5th year of follow-up, as described in Fig. 1.

In univariate logistic regression analyses, factors associated with the occurrence of cardiovascular disease events were an overexpression of HER-2 (HR 2.6 [1.21–5.56] *p* < 0.011) as well as UICC-stage III tumors or more (HR 5.47 [2.78–10.76] *p* < 0.001). Patients with pre-existing cardiovascular disease risk factors were at higher risk to develop cardiovascular disease events (HR 2.91 [1.36–6.23] *p* < 0.004). However, patients with metastatic breast cancer at diagnosis had no higher risk of developing cardiovascular disease events. Factors associated with the occurrence of cardiovascular disease events are summarized in Table 3.

**Table 3:** Factors associated with the occurrence of cardiovascular events

***N*** = 682	Modality	No Event	Event
HER2 overexpression (*N* = 682)	Y	551 (85%)	25 (73.5%)
	N	69 (10.6%)	9 (26.5%)
	NA	28 (4.3%)	0 (0%)
Left breast (*N* = 682)	Y	340 (52.5%)	17 (50%)
	N	308 (47.5%)	17 (50%)
	NA	0 (0%)	0 (0%)
Triple negative status (*N* = 682)	N	590 (91%)	32 (94.1%)
	Y	56 (8.6%)	2 (5.9%)
	NA	2 (0.3%)	0 (0%)
Metastatic at diagnosis (*N* = 682)	Inf IV	634 (97.8%)	33 (97.1%)
	IV	14 (2.2%)	1 (2.9%)
	NA	0 (0%)	0 (0%)
UICC (*N* = 682)	<III	573 (88.4%)	19 (55.9%)
	> = III	75 (11.6%)	15 (44.1%)
	NA	0 (0%)	0 (0%)
Chemotherapy with anthracyclines (*N* = 682)	Y	162 (25%)	12 (35.3%)
	N	73 (11.3%)	9 (26.5%)
	NA	413 (63.7%)	13 (38.2%)
Hormonotherapy (*N* = 682)	Y	525 (81%)	26 (76.5%)
	N	123 (19%)	8 (23.5%)
	NA	0 (0%)	0 (0%)
Pre-existing cardiovascular disease risk factors (*N* = 682)	N	332 (51.2%)	9 (26.5%)
	Y	315 (48.6%)	25 (73.5%)
	NA	1 (0.2%)	0 (0%)

## Discussion

### Main findings

In the present study, the 5-year cumulative incidence of cardiovascular disease following diagnosis of breast cancer was 5.8% ([3.8–7.7] (CI 95%). Several factors influence the rising incidence of cardiovascular disease in survivors of breast cancer. These include competing risk of ageing, shared risk factors for both cardiovascular disease and breast cancer as well as smoking or obesity, and the impact of breast cancer treatments [17]. Indeed, even if cardiovascular disease can be caused or accelerated by breast cancer treatments as for example heart failure caused by anthracycline chemotherapy and trastuzumab, it is the combination to direct cardiotoxic effects and indirect effects of cancer therapy, combined with patient factors such as pre-existing hypertension, diabetes, mellitus, dyslipidemia, or smoking that explains the growing burden of cardiovascular disease in breast cancer patients [18]. In the present study, 50% of the patients had pre-existing risk factors for cardiovascular disease, hence the need of cardiovascular disease risk stratification at the time of breast cancer diagnosis for prophylactic strategies among patients at higher risk. Most of the cardiovascular disease events occurred within the first year after diagnosis, with a secondary increase between the 4th and 5th year of follow-up, highlighting two intervals of time at risk. Our results are relevant as the absolute risk of dying of CVD following breast cancer ranges from 1.6 to 10.4% [19]. A recent study has pointed out that the cumulative incidence of CVD 20 years after breast cancer treatment was 11.3% for those who received radiotherapy with a cardiovascular risk factor at diagnosis, and it was higher if patients had received internal mammary chain irradiation [20].

According to the accumulated dose, the incidence of severe anthracycline-induced cardiotoxicity leading to systolic heart failure can be as high as 25% [21]. As regards trastuzumab, cardiotoxicity was initially high when it was given concomitantly with anthracyclines, in trials of metastatic breast cancer. The administration of trastuzumab after anthracyclines substantially reduced the rate of clinical heart failure, with a rate of cardiac dysfunction in patients treated with anthracyclines and trastuzumab of 6.2 and 20.1% after 1 and 5 years respectively [18]. The association between left-sided breast cancer and radiotherapy treatment with a higher risk of cardiovascular disease mortality has mainly been found in the early 1980’s as it usually involved higher doses on a large irradiation field. Risk of death from ischemic heart disease associated with radiation for breast cancer has substantially decreased over time [22].

### Diagnostic features

This analysis suggests that patients with breast cancer have a higher risk of cardiovascular disease events, and may represent a perfect panel of ‘long-term survivors’ from cancer, for which dedicated preventive strategies should be elaborated. Garcia M et al. have highlighted emerging, non-traditional risk factors including breast cancer treatments that contribute to increase the risk of cardiovascular disease among women, claiming for aggressive prevention strategies [23]. Indeed, patients with breast cancer who have received anthracycline-based therapy as well as mediastinal radiation therapy should be included in long-term cardiac surveillance programs. In the present study, factors associated with the occurrence of cardiovascular disease events are mainly patients with pre-existing cardiovascular disease risk factors as well as aggressive cancer factors such as the overexpression of HER2 and advanced stage of cancer. However, no multivariate analysis was done, because of the paucity of events. Hitherto, the only score developed to predict the risk of major adverse cardiovascular disease events among breast cancer patients mainly includes cardiovascular disease risk factors at the time of diagnosis. Specific breast cancer treatments associated with cardiovascular toxicity as well as relevant baseline variables such as tumor characteristics were not considered. Subsequently, our findings concerning the overexpression of HER2 and advanced stage of cancer that represent factors associated with the occurrence of cardiovascular disease may be implemented in such scores [24]. Almost 90% of the patients were alive after a 5 years follow-up while 30 patients (4.4%) had a breast cancer relapse. The American Heart association underlines that breast cancer survivors who are 65 and older are more likely to die of cardiovascular disease events than breast cancer, breast cancer increasing the risk of cardiovascular disease at the same time, hence the interest of omics signatures for slowing the therapeutic armamentarium and to tailor the appropriate treatment to each patient, according to their accurate cancer’s risk recurrence, and subsequently spare the heart [25]. Breast cancer survivors should be closely monitored as they may live long enough to be at risk for competing causes of death. In that regard, an effective screening tool for identifying cardiac damage in breast cancer survivors may not be too far off through the identification of bio markers that could lead to earlier detection of cardiac damage, subsequently allowing the instauration of cardioprotective therapies [26]. Trials are currently exploring how effective it may be to give these agents during postoperative chemotherapy and radiotherapy [27, 28].

### Focus on AF

Considering AF, the cumulative incidence was 1.1% [0.1–2.1] (CI 95%). Epidemiological evidence of AF in cancer patients, which has been initially mostly explored in colorectal and lung cancers through case-control studies, found prevalence rates of AF after cancer diagnosis ranging from 0.59 to 5.2% [29–31]. According to Guzetti et al.*,*the relation between AF and cancer does not seem to be restricted to particular cancer location, suggesting that cancer could lead to AF through a systemic inflammatory state [13]. A cohort study of 269,742 patients based on Danish registry data displayed that the relative risk of cancer diagnosis was high for all types of cancer within 3 months after AF diagnosis but especially pronounced for lung, kidney and colon cancers. Similarly, in a long-term prospective cohort study of 34,691 patients, the risk of incident AF after cancer diagnosis was 20% higher in the first 3 months and especially for colon cancer [32, 33]. Beyond systemic inflammation, AF may complicate the course of breast cancer, notably through local factors including thoracic radiation [34]. The development of AF also possibly represents a complication of medical breast cancer therapy as several chemotherapeutic agents are associated with AF arrhytmiogenesis [35]. Further research is needed to explore such hypotheses [24]. Nevertheless, even though breast cancer doesn’t seem to be the first provider of AF, as illustrated by our figure analysis, it should not be considered as an epiphenomenon, particularly in view of therapeutic issues.

### Therapeutic features of AF in breast cancer patients

Important dilemmas remain in the treatment of patients with new-onset AF occurring during cancer course concerning both antithrombotic and antiarrhythmic therapy. One major unresolved concern is the consideration of AF occurring during cancer course as a classical AF that would lead to the same conventional treatment. The present study showed that patients with AF were allocated treatments with either direct oral anticoagulants (DOACs) or low molecular weight heparins (LMWH). This reflects the fact that no clinical guidelines in the management of AF following cancer diagnosis are currently available, notably when antithrombotic treatment is chosen, the same for antiarrhythmic strategy. If DOACs are being increasingly prescribed for patients with AF in common population, data concerning DOACs in patients with AF and active cancer are scarce [36, 37]. The large clinical trials of dabigatran, apixaban, and rivaroxaban for stroke prevention in AF have excluded patients with active cancer [36, 38, 39]. If DOACs are non-inferior to LMWH for the treatment of venous thromboembolism in cancer patients, there is however a higher risk of major bleeding associated to their use [40, 41]. LMWH may be used preferentially, even if no sufficient data support the relevance of such therapeutic management. Indeed, the 2018 European Heart Rhythm Association Practical Guide on the use of non-vitamin K antagonist oral anticoagulants in patients with AF has addressed the scope of the issue and suggests a dedicated interdisciplinary team approach for assessing the accurate anticoagulant treatment in cancer patients with AF since further data are still required [42]. Trials are currently ongoing to notably demonstrate the efficacy and safety of apixaban in comparison to LMWH for treating venous thromboembolism among patients with cancer [43]. Finally, there is still no strong evidence to guide practice despite the fact that both AF and breast cancer have growing incidences.

### Limits

In the interpretation of our findings, some strengths and limitations deserve to be taken into account. The major strength of our study is of methodological nature. All patients from the area were treated in the same center, so we feel confident that any patient with diagnosed breast cancer has been screened and included in our analysis. Since the first national “Plan Cancer” promoted by Président Jacques CHIRAC in 2003, the management of patients with cancer has been devoted to the corresponding oncologist. Thereby, the risk of missing information due to the retrospective may be low. Moreover, existence of missing information would have increased the rate of cardiovascular disease events. So, we feel confident that the incidence of cardiovascular disease events after breast cancer diagnosis is at least 5.8% [3.8;7.7] 95%CI. Besides, as regards cardiovascular disease events, we had an access to cardiology files of Saint-Etienne University hospital center where patients are usually admitted for cardiovascular disease events. The occurrence of other cardiovascular disease events which could have been passed under silence was sought by calls to each patient’s general practitioner. The same process was used to collect pre-existing cardiovascular disease. Because of the retrospective collection of information, we then decided to proceed solely to an univariate analysis.

## Conclusion

This work sets the basis for the exploration of complex interactions between breast cancer and cardiovascular disease as they appear not to be an epiphenomenon. That should ultimately lead to the development of guidelines regarding the prevention and treatment of cardiovascular disease and AF in breast cancer, particularly among women. Collaborative efforts between cardiologists and oncologists must be stepped up to this aim. Future studies are needed to deeply explore this issue, especially concerning mechanisms underlying the association between AF and breast cancer therapies particularly by exploring the two main hypotheses (systemic inflammation and autonomic activity disequilibrium). Identification of these factors may have major public health implications for cancer prevention.

## Data Availability

data and material were available from patients’ files, with their written consent. All data generated or analyzed during this study are included in this published article.

## Abbreviations

**AF**: Atrial fibrillation
**VTE**: Venous thromboembolism
**UICC**: Union for international cancer control
**NCI/CTCAE**: Common terminology criteria for adverse events
**DOAC**: Direct oral anticoagulant
**PE**: Pulmonary embolism
**DVT**: Deep venous thrombosis
**SVT**: Superficial venous thrombosis
SD: Standard deviation
**LMWH**: Low molecular weight heparins
**SBR**: Scarff-bloom-richardson
**ACE**: Angiotensin-converting-enzyme
**AIIRAs**: Angiotensin II receptor antagonist
**CCB**: calcium channel blockers
**FEC**: 5 Fluoro-uracile, epirubicine, cyclophosphamide
**TAC**: Docetaxel, adriamycine, cyclophosphamide
